# Computational Anesthesiology: A New Frontier at the Intersection of Data Science and Perioperative Medicine

**DOI:** 10.5812/aapm-175132

**Published:** 2026-04-30

**Authors:** Firoozeh Madadi

**Affiliations:** 1 Department of Anesthesiology, Anesthesiology Research Center, School of Medicine, Ayatollah Taleghani Hospital, Shahid Beheshti University of Medical Sciences Tehran Iran

**Keywords:** Artificial Intelligence, Computational Intelligence, Machine Learning, Perioperative Management, Closed-loop Anesthesia, Digital Twins, Predictive Analytics, Precision Medicine

Dear Editor,

Computational medicine is an emerging discipline that uses computational simulations, computational models, and sophisticated software to simulate disease progression and treatment (1). Anesthesiology and perioperative care have long focused on real-time monitoring, rapid decision-making, and precise drug titration (2-4). Over the past decade, advances in computational power, machine learning, and high-resolution physiological data have created a distinct domain: computational anesthesiology. We propose formal recognition of this emerging field, supported by dedicated academic attention, research funding, and methodological scholarship.

Computational anesthesiology applies modeling, artificial intelligence (AI), signal processing, and data-driven algorithms to understand, predict, and optimize anesthetic care. Computational and data-driven approaches can also support mechanistic investigations relevant to anesthesia and pain. Experimental neuron models have been used to study N-methyl D-aspartate receptor-mediated activation (5), and network analysis has been applied to evaluate gene-expression changes associated with ropivacaine exposure (6). Mechanistic evidence of ketamine-related calcium homeostasis and mitochondrial dysfunction further illustrates the molecular-level data that may inform computational investigations in anesthesiology (7).

Key domains include:

1. Pharmacokinetic-pharmacodynamic modeling for individualized dosing that accounts for patient-specific factors.

2. Closed-loop systems that automatically adjust hypnotics, analgesics, and neuromuscular blockers using electroencephalography (EEG), nociception, or hemodynamic feedback (8).

3. Machine-learning risk prediction for hypotension, delirium, kidney injury, or mortality, often with actionable lead times (9).

4. Digital twins that simulate patient-specific responses for in silico testing of strategies (10).

5. Emerging brain-computer interface concepts that may extend computational approaches in regional anesthesia by linking neural signals with technology-assisted clinical applications (11).

6. Advanced neuromonitoring analyses that refine depth-of-anesthesia assessment beyond commercial indices (12).

7. Natural language processing to extract risk factors from unstructured records for quality improvement and research.

Three forces make formal recognition timely:

- High-fidelity intraoperative datasets now exceed the capacity of traditional analytic methods.

- Deep-learning architectures suited to time-series and multimodal data can extract meaningful patterns from this complexity.

- Multicenter perioperative databases are providing the scale needed for robust development and external validation.

Enthusiasm must be tempered by rigor. Many published models lack external validation, remain opaque, or are poorly calibrated across populations. Closed-loop systems raise questions about accountability. AI tools must be integrated carefully to avoid alert fatigue and preserve clinical judgment; they should augment, not replace, the anesthesiologist. Anesthesiology journals should advance the field by prioritizing multicenter external validation, requiring transparent reporting standards (TRIPOD+AI, CONSORT-AI), fostering interdisciplinary collaboration, and creating dedicated sections or special issues on computational perioperative care (13).

Computational anesthesiology is already reshaping bedside practice. Formal recognition, paired with methodological discipline, will help ensure that its advances are safe, generalizable, and clinically meaningful.
